# Reaching out to Patients with Long COVID to Better Understand Their Life Experiences and How to Support Their Recovery: A Patient-Oriented Knowledge Sharing Session

**DOI:** 10.3390/ijerph21020187

**Published:** 2024-02-06

**Authors:** Alexa Pommer, Gayle Halas, Rukmali Mendis, Cindy Campbell, Brenda Semenko, Brandy Stadnyk, Leyda Thalman, Susan Mair, Yue Sun, Neil Johnston, Diana C. Sanchez-Ramirez

**Affiliations:** 1 Department of Respiratory Therapy, Rady Faculty of Health Sciences, University of Manitoba, Winnipeg, MB R3E 0T6, Canada; 2 Winnipeg Regional Health Authority, Winnipeg, MB R3E 0M1, Canada; 3 Misericordia Health Centre, Winnipeg, MB R3C 1A2, Canada; 4 The Manitoba Lung Association, Winnipeg, MB R3A 1M5, Canada

**Keywords:** long COVID, community, patient experience, healthcare

## Abstract

This article reports on participants’ experiences with long COVID-19 (LC) (symptoms, impact, healthcare use, and perceived needs) and satisfaction with a patient-oriented knowledge-sharing session organized by a multidisciplinary team of healthcare professionals, researchers, and a patient partner. Twenty-six participants completed a pre-session survey. On average, they were 21 months post-COVID-19 infection (SD 10.9); 81% of them were female, and 84% were 40+ years old. The main symptoms reported included fatigue (96%), cognitive problems (92%), and general pain or discomfort (40%). More than half of the participants reported that LC has had a significant impact on their health-related quality of life. Eighty-one percent of the participants reported seeking medical help for their LC symptoms and found the services provided by physical therapists, primary care providers, and acupuncturists to be helpful in managing their condition. Participants would like to have access to healthcare providers and clinics specializing in LC. They liked the session and found the information presented useful. This information helps to better understand the experiences of people living with LC and how to support their recovery.

## 1. Introduction

There have been over 769 million Coronavirus Disease 2019 (COVID-19) infections and 6.9 million deaths worldwide as of 9 August 2023 [1]. More than three and a half years have passed since the pandemic initiated a crisis response to a novel health threat. While an increase in knowledge of the virus and related vaccine discoveries have alleviated the acute nature of the pandemic, many individuals still suffer from a range of sequelae from the virus. Long-COVID (LC) is a condition characterized by persistent or new symptoms associated with the SARS-CoV-2 virus present 3 months or more after the initial infection that cannot be explained by an alternative diagnosis [2]. It is estimated that at least 10–20% of people infected by the virus may experience LC symptoms, which can be severe enough to hinder return to daily activities [3]. With over 200 different symptoms reported, LC can have vastly different presentations among people, and in combination with a lack of formal testing, this presents challenges for proper diagnosis and management [4]. Consequently, resources to support LC management are often limited, and patients may sometimes be unaware of the local alternatives available to them. Due to the novelty and variability of this condition, there is no consensus on how to support patients nor on the allocation of adequate resources and the care pathways needed within the healthcare system [5].

Several studies exploring LC have been conducted primarily using medical records or information collected from healthcare users [6,7]. Consequently, healthcare data may not fully capture the physical and emotional burdens caused by this condition and the difficulty of navigating a system that cannot adequately address the issues. In one of our recent survey-based studies, about 16% of people with LC reported that they did not seek medical care due to COVID-19 or LC symptoms [8]. Although milder symptoms could explain the lack of use of health services, between 5 and 11% of these patients reported reduced daily activities due to LC symptoms, suggesting that it is necessary to explore other potential reasons behind the non-utilization of medical care in this group of patients [8]. Furthermore, studying the experiences of LC patients who have not used medical services would help to obtain a more accurate and comprehensive picture of the burden of this condition.

To better understand the experiences of people living with LC and how to support their recovery, it is essential to connect with them and hear their voices through different channels beyond formal research contexts [9]. This could potentially be achieved by providing a safe space for the exchange of information and experiences among LC patients, healthcare professionals, and researchers. Therefore, we organized a patient-oriented session to provide (1) an overview of the current long COVID knowledge and local resources available to manage it, and (2) an opportunity to share and discuss participants’ experiences and perceived needs. This article reports on participants’ experiences with LC and satisfaction with the session.

## 2. Methods

### 2.1. Session Planning

A multidisciplinary team of healthcare professionals involved in the care of patients with LC (physical therapist, occupational therapist, respiratory therapist, and a social worker), LC researchers, and a patient partner living with LC convened to plan and participate in the session. Two planning meetings took place to determine the itinerary and approach of the session. Invitation posters were designed, shared on social media (Facebook, LinkedIn, and Twitter), and distributed by email. Participants registered using an online platform to attend the event free of charge.

### 2.2. Pre-Session

Participants were sent a link to a short anonymous survey (Slido platform) via the registration platform and were informed that the anonymous information collected was to be presented during the session. A paper version of the survey was also made available at the beginning of the session (on-site) to collect information from people who were unable or unwilling to complete it online. The survey collected data on the following: demographics (sex, age), first COVID-19 infection (month/year), most bothersome symptoms selected by the participants from a list compiled based on existing literature, and impact on overall health-related quality of life using the EQ-5D-5L [10]. This is a validated measurement tool that has two components: a descriptive system with 5 dimensions (mobility, self-care, usual activities, pain/discomfort, anxiety/depression) and a visual analog scale for self-rated health (0 being the worst, 100 being the best) [10]. Additionally, information on healthcare services or providers accessed (and the perceived benefit) and resources or programs they wish to see available were also collected. The responses gathered from the paper surveys were not included in the presentation, as there was not sufficient time to transcribe them into the electronic version. Even if these responses were not shared, having this option available gave participants an opportunity to reflect and share their experiences when they would have otherwise not been able to do so. The data gathered from this survey (both versions) will be presented in Section 3 of this paper.

### 2.3. Session

The session took place on 27 June 2023 (~2 h) and contained several presentations delivered by different members of the team. The presentations included: (1) an overview of the available knowledge surrounding LC (prevalence, potential causes), LC’s impact on patients’ health and daily activities (reported in the literature and by the participants of the session), and LC healthcare use and perceived needs (reported by the participants of the session); (2) information about self-management resources for mild to moderate symptoms (Shared Health Provincial LC Website); (3) self-management virtual education sessions (Winnipeg Regional Health Authority Self-Management Group); and (4) a local LC rehabilitation program (Easy Street Program at Misericordia Hospital). The information collected from the pre-session survey was displayed during the first presentation of the session. This provided participants with the opportunity to relate their symptoms and/or experiences with the literature and with one another. Participants had the opportunity to ask questions and share their opinions and/or experiences throughout the sessions, with the aim of creating connections among the attendees and the ideas and concepts presented.

### 2.4. Post-Session

Following the session, all participants were sent another link via the registration platform to complete a post-session feedback survey. Participants were asked to rate the session and usefulness of the information presented, if they would like to attend a similar activity in the future, and what type of information they would like to see included.

## 3. Results

### 3.1. Session

Registration for the session was set up on Eventbrite and was open to everyone, including family members or others caring for someone with LC. Out of the 41 people who registered for the session, the majority were people experiencing LC (73%), followed by family members (15%), and others who had no personal connection to LC (12%).

In total, 30 people attended the session, including 28 who registered in advance and 2 who presented without having registered. Fourteen participants evaluated the session in an online post-session survey. Overall, the participants liked the session (mean 4.29 out of 5 (SD 0.73), found the information presented useful (mean 4.15 out of 5 (SD 0.8)), and 93% would like us to host a similar event in the future.

### 3.2. Patients’ Experiences with LC

People who registered were asked to complete a pre-session survey if they were experiencing long COVID symptoms using the following sentence, “we would greatly appreciate if the person experiencing long COVID completes this short survey prior to the meeting”. Twenty-six people completed the pre-session survey (20 online, and 6 filled out a paper version at the start of the session). The small discrepancy between the number of people registered and the pre-session surveys completed may be explained by the fact that some of the people who completed the online survey may not have been able to attend the session, and some of the attendees may have been family members or other caregivers who did not need to complete the survey.

The survey was completed mostly for females (81%), over the age of 41 (84%), and a mean of 21 months post-COVID-19 infection (SD 10.9). Participants’ most bothersome symptoms included fatigue (96%), cognitive problems (92%), and general pain or discomfort (40%) (Table 1). Fifty-eight percent of the participants reported moderate or severe issues with mobility, but only 15% had moderate or severe difficulty with self-care. All but one participant reported issues with performing their usual activities, with 81% experiencing moderate to extreme problems. Sixty-one percent of participants experienced moderate or severe levels of pain or discomfort, and 46% experienced anxiety and/or depression related to their LC. The mean self-reported health status was 47.3 out of 100 (SD 15.9).

Eighty-one percent of the participants reported seeking medical help for their LC symptoms (Table 2). The most popular healthcare providers consulted were family doctors or nurse practitioners (90%), followed by specialists (71%) and physical therapists (52%). Two-thirds of the participants (67%) indicated they had consulted other healthcare providers, such as acupuncturists, chiropractors, massage therapists, social workers, and dieticians, or accessed programs to deal with their LC symptoms. Some participants found the services provided by primary care providers (3/19), physical therapists (4/11), and acupuncturists (3/4) to be quite helpful in improving or managing their condition. In addition, participants reported that being believed and having their symptoms taken seriously by providers was an aspect that was especially supportive. Conversely, 9 out of 21 of the participants who had sought medical care indicated that they had encountered at least one healthcare provider who was not responsive to their ongoing symptoms.

Some of the main resources the participants would like to have available to support their recovery include a healthcare provider that specializes in LC (88%), a clinic that specializes in LC (80%), more advertising of the local resources available (48%), more primary care providers aware of LC (44%), and a local support group (40%). Participants also expressed wanting resources to help with cognitive issues and fatigue, recognition of LC as a medical condition, better access to comprehensive healthcare services, an exercise regime specific to LC, information about job searches and/or accommodation, and financial/disability assistance for those living with LC.

## 4. Discussion

To the best of our knowledge, this is the first report on an LC patient-oriented knowledge-sharing session planned and executed by a multidisciplinary team and a patient partner. The session was positively received by patients and provided an opportunity to compile information about the experiences, impact, healthcare use, and perceived needs of patients living with LC. These findings may help healthcare providers and researchers better understand the burden of the condition, as they capture information from people who may not be interested in participating in formal research studies or who may not have been in contact with the health system and, therefore, could not be included in patient cohorts or identified in medical records. Almost one out of five participants in this session reported that they did not seek medical help for their LC symptoms. Despite the small sample size, this result aligns with what we found in a previous study in which 18.9% of participants with LC (223/1178) reported not seeking care due to their COVID-19 or LC symptoms [8]. In Canada, health care delivery is a provincial/territorial responsibility whereby hospitals, emergency departments, primary care, and specialist services are publicly funded. Physiotherapists, occupational therapists, respiratory therapists, and speech therapists can be accessed at private fee-for-service clinics or by referral and are not always covered by health insurance, which may limit access to these services. Additionally, LC clinics comprised of interdisciplinary healthcare teams are currently available in some Canadian provinces but not in Manitoba. Some of the reasons why certain people with LC do not seek medical care for their LC symptoms may include being unable to access primary care, accessing primary care but receiving (perceived) inadequate support, or alternatives to mainstream healthcare being available [11]. Those causes may be more pronounced among members of BIPOC (black, indigenous, and other people of color) and immigrant communities who have been systematically affected by structural inequities in healthcare [12]. However, more research is needed on the barriers to care faced by LC patients of different socioeconomic characteristics and racial and ethnic backgrounds and how to overcome them in the Canadian context.

The information shared by the participants aligns with existing evidence regarding the most common symptoms of LC and their detrimental impact on patients’ quality of life [13,14]. Some participants identify healthcare providers and services to be helpful in improving or managing their condition. Unfortunately, due to the vast range of symptoms that people experience, the services, providers, and treatments perceived as effective also vary between individuals [15]. Participants also indicated they consulted alternative health providers (acupuncturists, naturopaths, osteopaths, etc.), tried over-the-counter medications (Ibuprofen, Acetaminophen, Naproxen, muscle relaxants, etc.), and took vitamins and/or other supplements. This aligns with previous evidence suggesting that some LC patients are attempting to self-manage their symptoms, including the use of over-the-counter medications, supplements, therapies, and diets [16]. These findings allude to ongoing clinical uncertainty and lack of consensus about adequate management strategies for LC.

In addition to care resources to support their recovery, some participants expressed interest in receiving financial assistance and information on how to find employment suitable to their condition. Unfortunately, many people living with LC are unable to work due to their symptoms and have their application for disability benefits denied. Since there is no specific test for diagnosing LC, it is difficult to prove that someone has the condition, at least by the existing standards of insurance companies and Social Security. The lack of official guidelines has also resulted in many doctors being hesitant to support disability benefit applications. As a result, LC patients may experience a significant financial burden that must also be considered when providing comprehensive management to this group of patients.

Interestingly, 12% of the session attendees were not personally experiencing or caring for a family member with LC symptoms. Based on the email address used for the registration on the event platform and a few direct inquiries to the organizing team about the session, we speculate that some of these people could be healthcare providers who were seeking to be informed. Although we cannot be certain, this suggests that care providers may be interested in learning more about the condition, the local resources available, and how to best support the people in their care. This scenario would suggest an appeal for involving these stakeholders in LC research and education initiatives, including continuing professional development.

### 4.1. Lesson Learned and Future Considerations

Despite allocating sufficient time for discussion and breaks during the planning of the activity, the presentations and discussions ran longer than expected. It was tiring for some of the participants, and unfortunately, a few of them needed to leave before the end of the session. This is a lesson that can be applied to future sessions, where the presentations should be shorter, and more time should be allocated for participants to interact with one another.

### 4.2. Limitations

It is important to take into account that this report is based on an intervention originally conceived as a patient-oriented knowledge-sharing session and not as a research study. The data elicited from the participants were to inform an initial exploration of LC experiences and perceived needs. As such, some limitations should be considered when interpreting the results. First, the sample was not purposefully identified. Although the team intended to advertise the event widely, it may not have reached all potential participants, which could have introduced a selection bias. Second, information was not collected on some important characteristics of the participants, such as ethnicity, comorbidities, pre-COVID-19 health, occupation, or socioeconomic status. We were cautioned and careful to structure the session and activities in a way that would not overburden the participants; thus, the survey only asked for responses to two demographic questions. Third, we did not collect information related to recurrent COVID-19 infections, which present as an LC risk factor. Evidence suggested an increased risk of LC after a second or third infection. However, since the exploration of LC risk factors was outside the scope of this report, the lack of this information does not affect the findings reported in this manuscript. Fourth, a small number of people participated in the session. However, we believe the process and findings are valuable to inform future events and research involving a larger group of participants.

## 5. Conclusions

This patient-oriented knowledge-sharing session was positively received by the participants and provided an opportunity to compile information about the experiences, impact, healthcare use, and perceived needs of patients living with LC. The findings add to the growing body of knowledge aimed at better understanding the burden of the condition, as they capture information from people who may not be interested in participating in formal research studies or who may not have been in contact with the health system. Future planning of these activities should consider shorter presentations, extra breaks, and more time for interaction between participants.

## Figures and Tables

**Table 1:** Long COVID patient characteristics.

Variable	*n* = 26
Gender (*n*, % female)	21 (81%)
Age group (*n*, %)	
21–30	2 (8%)
31–40	2 (8%)
41–50	5 (19%)
51–60	10 (38%)
>60	7 (27%)
Months since first COVID-19 infection, mean (sd)	21 (10.9)
Most bothersome symptoms *	
Fatigue	24 (96%)
Cognitive problems (memory loss/difficult thinking or concentrating)	23 (92%)
General pain/discomfort	10 (40%)
Shortness of breath	9 (36%)
Mental health symptoms (such as anxiety or depression)	5 (20%)
Heart palpitations	2 (8%)
Chest pain or tightness	1 (4%)
Trouble sleeping	1 (4%)
Health-related quality of life (EQ-5D-5L)	
Issues with mobility	
None	3 (12%)
Slight	8 (31%)
Moderate	12 (46%)
Severe	3 (12%)
Extreme	0
Issues with self-care	
None	13 (50%)
Slight	9 (35%)
Moderate	4 (15%)
Severe	0
Extreme	0
Issues with performing usual activities (e.g., work, housework, family, or leisure activities)	
None	1 (4%)
Slight	4 (15%)
Moderate	16 (62%)
Severe	4 (15%)
Extreme	1 (4%)
Levels of pain or discomfort	
None	3 (12%)
Slight	7 (27%)
Moderate	12 (46%)
Severe	4 (15%)
Extreme	0
Mental health symptoms (depression/anxiety)	
None	3 (12%)
Slight	11 (42%)
Moderate	10 (38%)
Severe	2 (8%)
Extreme	0
Self-rated health status (0–100 best), mean (sd)	47.3 (15.9)
* Participants were asked to rank the 3 main symptoms. Some participants did not answer all the questions.	

**Table 2:** Healthcare use associated with long COVID.

Variable	*n* = 26
Have you been seen by a healthcare provider or participated in a program to manage your long COVID symptoms? (Select all that apply):	
No	5 (19%)
Yes	21 (81%)
Family doctor or nurse practitioner	19 (90%)
One or more specialists	15 (71%)
Physical therapist	11 (52%)
Occupational therapist	7 (33%)
Respiratory therapist	5 (24%)
Psychologist	2 (10)
Other providers/programs:	14 (67%)
Acupuncturist	4 (19%)
Chiropractor	2 (10%)
Massage therapist	3 (14%)
Social worker	1 (5%)
Dietician	1 (5%)
Naturopath	2 (10%)
Osteopath	1 (5%)
Long COVID group/rehabilitation	3 (14%)
Long COVID clinic/specialist	4 (19%)
Long COVID research study	2 (10%)
Have you encountered a provider who was not responsive to ongoing symptoms *n* = 21	
Yes	9 (43%)
No	12 (57%)
Some participants did not answer all the questions. Many selected more than one.	

## Data Availability

Data are contained within the article.
